# Identification of Unique mRNA and miRNA Expression Patterns in Bone Marrow Hematopoietic Stem and Progenitor Cells After Trauma in Older Adults

**DOI:** 10.3389/fimmu.2020.01289

**Published:** 2020-06-24

**Authors:** Dijoia B. Darden, Julie A. Stortz, McKenzie K. Hollen, Michael C. Cox, Camille G. Apple, Russell B. Hawkins, Jaimar C. Rincon, Maria-Cecilia Lopez, Zhongkai Wang, Eduardo Navarro, Jennifer E. Hagen, Hari K. Parvataneni, Maigan A. Brusko, Michael Kladde, Rhonda Bacher, Babette A. Brumback, Scott C. Brakenridge, Henry V. Baker, Christopher R. Cogle, Alicia M. Mohr, Philip A. Efron

**Affiliations:** ^1^Department of Surgery, University of Florida College of Medicine, Gainesville, FL, United States; ^2^Department of Molecular Genetics and Microbiology, University of Florida College of Medicine, Gainesville, FL, United States; ^3^Department of Biostatistics, University of Florida, Gainesville, FL, United States; ^4^Department of Orthopaedics, University of Florida College of Medicine, Gainesville, FL, United States; ^5^Department of Biomedical Engineering, University of Florida College of Medicine, Gainesville, FL, United States; ^6^Department of Pathology, Immunology and Laboratory Medicine, University of Florida College of Medicine, Gainesville, FL, United States; ^7^Department of Hematology and Oncology, University of Florida College of Medicine, Gainesville, FL, United States

**Keywords:** hematopoietic stem and progenitor cell, bone marrow, trauma, age, transcriptome, RNA, microRNA

## Abstract

Older adults have significantly worse morbidity and mortality after severe trauma than younger cohorts. The competency of the innate immune response decreases with advancing age, especially after an inflammatory insult. Subsequent poor outcomes after trauma are caused in part by dysfunctional leukocytes derived from the host's hematopoietic stem and progenitor cells (HSPCs). Our objective was to analyze the bone marrow (BM) HSPC transcriptomic [mRNA and microRNA (miR)] responses to trauma in older and younger adults. BM was collected intraoperatively <9 days after initial injury from trauma patients with non-mild injury [ISS ≥ 9] *or* with shock (lactate ≥ 2, base deficit ≥ 5, MAP ≤ 65) who underwent operative fixation of a pelvic or long bone fracture. Samples were also analyzed based on age (<55 years and ≥55 years), ISS score and transfusion in the first 24 h, and compared to age/sex-matched controls from non-cancer elective hip replacement or purchased healthy younger adult human BM aspirates. mRNA and miR expression patterns were calculated from lineage-negative enriched HSPCs. 924 genes were differentially expressed in older trauma subjects vs. age/sex-matched controls, while 654 genes were differentially expressed in younger subjects vs. age/sex-matched control. Only 68 transcriptomic changes were shared between the two groups. Subsequent analysis revealed upregulation of transcriptomic pathways related to quantity, function, differentiation, and proliferation of HSPCs in only the younger cohort. miR expression differences were also identified, many of which were associated with cell cycle regulation. In summary, differences in the BM HSPC mRNA and miR expression were identified between older and younger adult trauma subjects. These differences in gene and miR expression were related to pathways involved in HSPC production and differentiation. These differences could potentially explain why older adult patients have a suboptimal hematopoietic response to trauma. Although immunomodulation of HSPCs may be a necessary consideration to promote host protective immunity after host injury, the age related differences further highlight that patients may require an age-defined medical approach with interventions that are specific to their transcriptomic and biologic response. Also, targeting the older adult miRs may be possible for interventions in this patient population.

## Introduction

Traumatic injury remains one of the leading causes of morbidity and mortality in the United States and the world, despite advances in the management of these patients (1, 2). The majority of trauma patients are known to recover rapidly; however, 3-year mortality remains ~16%, and about 20% of trauma patients develop chronic critical illness (CCI) (3–6), defined as > 14 days in ICU with continued organ dysfunction (7–9). Importantly, studies have revealed that risk factors for poor post-traumatic outcomes include age and injury severity score (ISS) at admission as well as total blood transfusion in the first 12 h after injury (3, 10–13).

A dysfunctional host immune system is responsible in part for post-traumatic morbidity and mortality (8). This includes innate immune cells originating from the host's hematopoietic stem and progenitor cells (HSPC) in the setting of unresolving organ failure—leukocytes derived from these HSPCs are not able to adequately resolve secondary, post-traumatic infectious insults (8, 13–15).

Importantly, advanced age is associated with an attenuated acute peripheral leukocyte response and age is known to be one of the strongest risk factors for poor outcome after severe trauma with hemorrhagic shock (6, 11, 16). Older adults have a baseline dysfunction in their immune system (immunosenescence) and low grade systemic inflammation (inflammaging) that in part can contribute to their suboptimal immune response to inflammation, increasing poor outcomes after severe injury (17). Our laboratory, as well as others, have demonstrated that elderly mice after severe injury are unable to mount an effective immune response as compared to juvenile mice. This is secondary, in part, to a failure of bone marrow progenitors to effectively respond to trauma (18). In a murine trauma model, this resulted in increased mortality with delivery of post-trauma *Pseudomonas pneumonia* (18–21).

Numerous attempts at pharmacological and therapeutic interventions to prevent or attenuate post-traumatic insults in this high risk group of older adults have not sufficiently taken into account their unique response to severe injury (22–25). This includes the HSPC response to trauma, as well as the epigenome that in part controls the transcription of these host cells. Specifically, microRNAs (miRs), a class small, non-coding RNAs that regulate gene expression, are important to cellular transcriptional/epigenetic modification of multiple processes such as cell development and differentiation (26). miRs can functions in several ways, including RNA silencing and post-transcriptional regulation of gene expression, and are known to modulate immune responses (27). Modulating miRs has emerged as powerful therapeutic target for cell specific therapy in personalized medicine (28).

We hypothesized that the bone marrow (BM) HSPC transcriptomic and miR response to trauma would be dependent upon the age of the subject and could in part explain the post-trauma dysfunctional myelopoiesis in these individuals, and reveal potential therapeutic targets.

## Methods

### Study Approval

Approval was obtained from the University of Florida (UF) Institutional Review Board and was performed from 2014 to 2019 at UF Health Shands Hospital, a 996-bed academic quaternary-care referral center. The study was registered with *clinicaltrials.gov* (*NCT02577731*) and conducted by the Sepsis and Critical Illness Research Center at UF. In every case, signed, informed consent was obtained from the individual patient or their designated legal representative. If informed consent was obtained from the legal representative, the patient was re-consented after they had achieved a clinical state where they could provide informed consent. If written informed consent could not be obtained from the patient or their legal representative within 96 h of study enrollment, the patient was removed from the study and all collected biologic samples and clinical data were destroyed.

### Cohort Selection

We enrolled adult trauma patients (age ≥18) that were admitted to UF Health Shands Hospital according to the UF Institutional Review Board protocol #201601386. Patients with blunt and/or penetrating trauma resulting in long bone or pelvic fractures requiring open reduction and internal fixation or closed reduction, percutaneous pinning) were selected if they demonstrated an injury severity score (ISS) ≥ 9 *or* hemorrhagic shock (HS; defined by systolic blood pressure ≤ 90 mmHg or mean arterial pressure ≤ 65 mmHg or base deficit (BD) ≥ 5 meq or lactate ≥ 2) within the first 24 h of admission. Patients were excluded if pregnant, prisoners, expected survival was <48 h, receiving chronic corticosteroids or immunosuppression therapies, previous bone marrow transplantation, previous diagnosis of End Stage Renal Disease, or any pre-existing hematological disease.

Age/sex-matched controls were either purchased whole human bone marrow (Lonza, Biosciences, Berkshire, U.K.) or bone marrow collected from non-cancer, non-infectious elective hip repair patients. Control patients were enrolled according to the same IRB protocol above and were excluded if pregnant, prisoners, receiving chronic corticosteroids or immunosuppression therapies, previous chemotherapy or radiation therapy, previous bone marrow transplantation, previous diagnosis of end stage renal disease, or any pre-existing hematological disease, pathological fractures, cancer, HIV or connective tissue disease.

### Bone Marrow Collection

A 10 ml aspirate sample of whole bone marrow was collected intraoperatively. Of note, this aspirate contained tissue and not just cellular material. This tissue was considered a waste sample as it would have to be removed to make room for the hardware being placed by the orthopedic surgeons. Bone marrow was collected into a heparinized tube, placed on ice and transferred to the laboratory and processed <12 h after collection (29).

### HSPC Transcriptomic Profile Analysis

Bone marrow collection and bone marrow cell isolation were conducted as previously published by our laboratory (29). HSPCs from either trauma, control or purchased whole human bone marrow were negatively isolated from bone marrow via magnetic separation using a lineage-positive, cell depletion kit according to protocol (Miltenyi Biotec). Total RNA was isolated using QIAGEN RNeasy™ Mini Kit (Qiagen) and labeled and hybridized onto GeneChip® Human Transcriptome Array 2.0 (Affymetrix, Santa Clara, CA) and processed following manufacturer's instructions. BRBArray Tools® was used to identify significant microarray gene expression differences. Fold expression changes of the significant genes were calculated vs. age/sex-matched controls. Three separate analyses were performed based on: (1) Patient age: younger <55 years or old ≥ 55 years; (2) ISS score; and (3) blood transfusion.

The significant, differentially expressed genes were further analyzed with Ingenuity Pathway Analysis (IPA) software™ and Gene Ontology™ (GO) enrichment analysis. IPA software was employed to make downstream functional predictions from these groups of genes with a Z-score greater than two indicating significance. GO enrichment analysis identifies significant representative pathways/biogroups that are over-represented, indicating that their expression is influenced by the intervention. We selected pathways with *p* ≤ 0.005, determined by the LS/KS permutation test and Efron-Tibshirani's GSA maxmean test utilized in GO software to find significant gene sets.

### miRNA Expression and miRNA Target Gene Prediction

miRNA (miR) profiling was performed using GeneChip™ miRNA 4.0 Array (ThermoFisher Scientific), covering 2,578 human microRNAs annotated in miRBase V2.0. miR expression patterns were calculated with a log_2_-transformed expression matrix with significant expression differences (fold expression changes over age/sex-matched control) identified using BRBArrayTools® (*p* < 0.05). Predicted target genes of the differentially expressed miR were identified with TargetScan Human 7.2, which predicts biological targets of miRs, by searching for conserved 8-mer, 7-mer and 6-mer sites matching the seed region of each miR (30).

### Statistics

Results for continuous variables are reported as mean ± SD for normally distributed variables or median ± interquartile range for non-normally distributed variables. Normality was checked via the Shapiro-Wilk test. Student's *t*-test or nonparametric Mann-Whitney test was used to compare normal or non-normal variables respectively between different groups or time points. Tukey's multiple comparison procedure was used to adjust *p*-values for multiple comparisons. Data were analyzed using Prism 7 (GraphPad Software, CA) and SAS 9.4 (SAS Institute Inc., Cary NC).

## Results

### Patient Characteristics

The overall and age-defined characteristics of the younger and older adult trauma patient cohorts of this study are displayed in Table 1. Previous studies have demonstrated that age ≥55 years is associated with worse outcomes after severe trauma (11, 31, 32). Younger and older adult groups were relatively similar with the exception of a higher percentage of trauma-related falls in the older group, as opposed to zero in the younger adult group. ISS, lactate, blood transfusion, and Apache II score from the first 24 h from admission did not significantly differ between the two groups (*p* = 0.48, *p* = 0.64, *p* = 0.85, and *p* = 0.58, respectively) (33–36).

**Table 1:** Patient characteristics.

	**Older ≥ 55 (*n* = 8)**	**Younger <55 (*n* = 25)**
Age, median (Q1, Q3)	62.5 (59, 65.75)	37 (28.5, 50)
Percent Male (%)	50	64
**Race**		
White (%)	87.5	76
African American (%)	12.5	24
**Mechanism of injury**		
Motor Vehicle Crash (%)	32.5	72
Motor Cycle Crash (%)	12.5	16
Pedestrian vs. Car (%)	12.5	12
Fall (%)	12.5	0
ISS, median (Q1, Q3)	24 (19, 31.5)	18 (15.5, 31.5)
Lactate, median (Q1, Q3)	3.05 (2.23, 3.71)	2.68 (1.96, 3.46)
MAP, median (Q1, Q3)	80.5 (68.75, 91.5)	81 (74, 91.5)
Days to Surgery, median (Q1, Q3)	4.5 (2, 7.5)	2 (1.5, 4.5)

Trauma bone marrow samples were obtained during long bone or pelvic fracture repair <9 days following blunt trauma (3.5 ± 2.4 days), and control samples (mean age 60.0 ± 9.1 years) were obtained at the time of elective surgery or from a commercial vendor (mean age 28.3 ± 4.4 years).

### Hematopoietic Stem and Progenitor Cells Genomic Analysis

**Figure 1:**
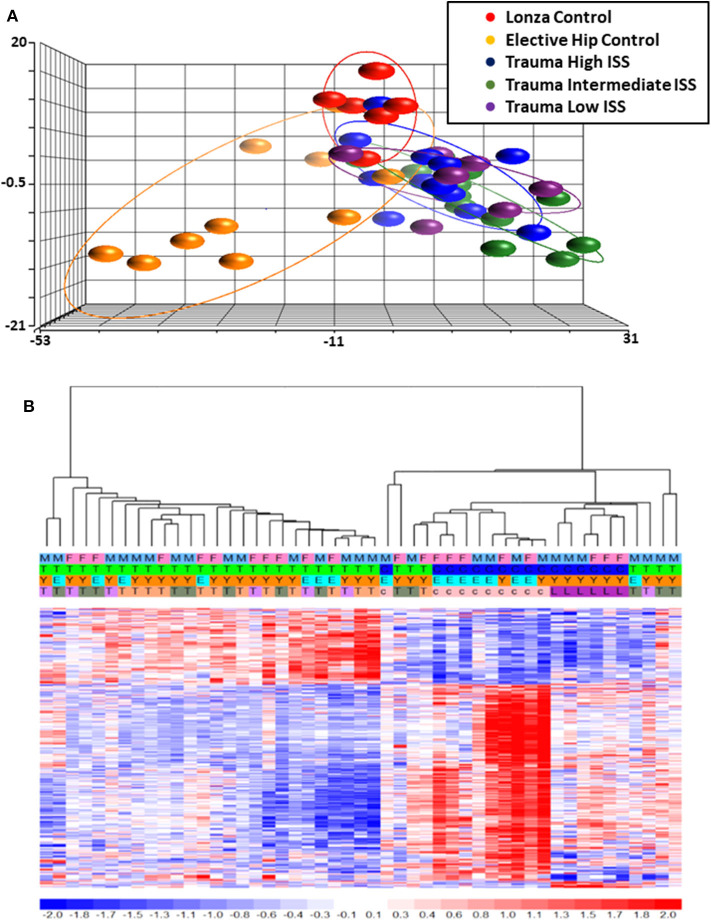
Microarray Transcriptomic Analysis of Leukocytes from Trauma Patients with Low, Intermediate and High ISS and Healthy Control Subjects. The genomic response of isolated leukocyte RNA in healthy controls and trauma patients and healthy controls. **(A)** Conditional principal component analysis of ISS and healthy control leukocyte gene expression patterns. **(B)** Heat map (log_2_) of the hierarchical clustering of leukocyte gene expression patterns and variation between trauma patients with differing ISS healthy control subjects. M, Male; F, Female; Y, Younger group; E, Older group; T, Trauma subject (three colors on row four represent three different ISS groups); c, Elective hip control subject; L, Lonza control subject.

**Figure 2:**
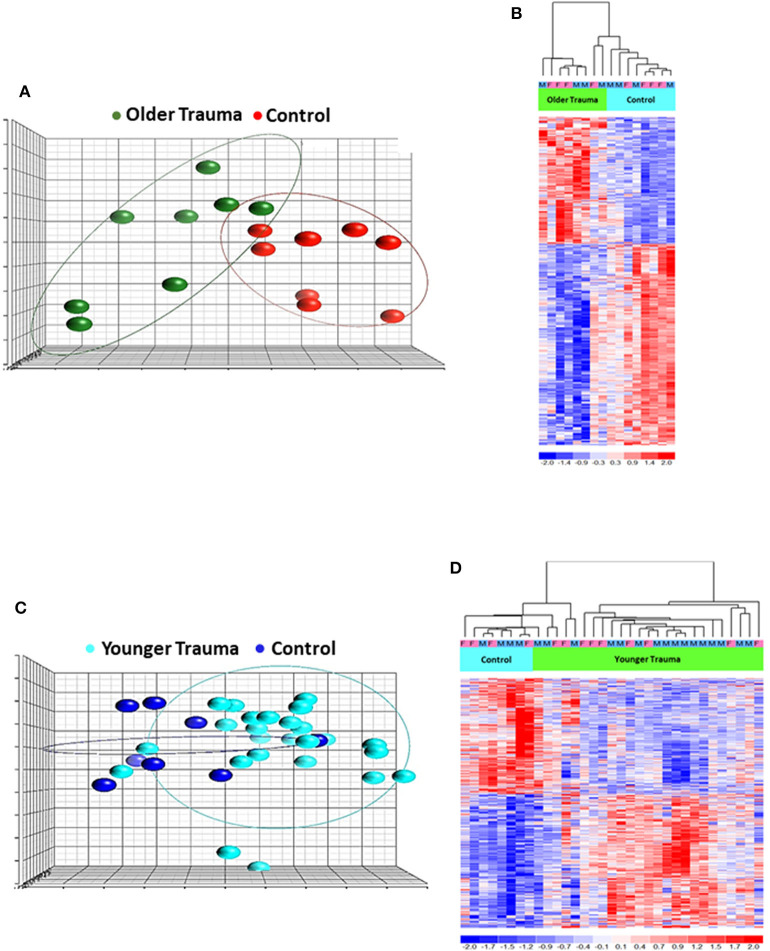
Microarray Transcriptomic Analysis of Leukocytes from Older Trauma Patients and Younger Trauma Patients vs. Healthy Control Subjects. The genomic response of isolated leukocyte RNA in healthy controls and trauma patients and healthy controls. **(A)** Conditional principal component analysis of older adult trauma patients and healthy control leukocyte gene expression patterns. **(B)** Heat map (log_2_) of the hierarchical clustering of leukocyte gene expression patterns and variation between older adult trauma patients and healthy control subjects. **(C)** Conditional principal component analysis of younger adult trauma patients and healthy control leukocyte gene expression patterns. **(D)** Heat map (log_2_) of the hierarchical clustering of leukocyte gene expression patterns and variation between younger adult trauma patients and healthy control subjects. M, Male; F, Female.

Total RNA was isolated from negatively-isolated bone marrow HSPCs for transcriptomic analysis. In an analysis of all trauma patients (*n* = 33) vs. healthy controls (*n* = 16), 1,845 genes were differentially expressed by enriched HSPC populations (*p* < 0.001). Sub-group analysis revealed that the severity of injury (ISS categories) did not significantly influence HSPC genome-wide expression (*p* = 0.083) (Figure 1). Transcriptomic differences were also not detectable between patients with or without blood transfusion (*n* = 7 and 26, respectively) within 24 h after injury. However, HSPC genome-wide expression did vary after trauma in older vs. younger adult patients. Older adult trauma patients (vs. age-matched controls) had a total of 924 probe sets representing 749 unique genes that were differentially expressed (*p* < 0.005; Figures 2A,B). Analysis of HSPCs in younger trauma vs. age-matched controls, however, revealed differential expression of 709 probe sets representing 654 unique genes (*p* < 0.0005; Figures 2C,D).

Interestingly, we determined that the majority of genes with significant up or down regulation after trauma (vs. control) were dissimilar between the older and younger adult trauma patients (Table 2). Of the 749 and 654 genes identified after trauma in older and younger adult trauma patients, respectively, only 68 genes were observed to be in common (~10%). In this set of common genes between older and younger adult trauma, the directionality of the change was identical, implying their importance to the common mammalian response to severe injury. Fifty-six genes were found to be downregulated, while the remaining 12 genes were upregulated (Table 3). Many of these genes are known to be important in inflammation and innate immunity, such as *MYCT1, NRIP1, FLT3, TNFAIP2, and BCL2* (37–40).

**Table 2:** Top 10 genes with the greatest significant expression changes in only older or younger adult trauma patients (relative to/vs. age-matched controls) in bone marrow HSPCs.

**Expression**	**Genes**
Upregulated in older trauma patients	*TREM1, RGS2, AQP9, PI3, LY96, GZMA, FCGR3B, TNFAIP6, IGSF6, IL1R2*
Downregulated in older trauma patients	*LMAN1, EEF2, CD34, LRBA, HSP90AB1, CSNK2A2, RSL1D1, MIR4737, SPTBN1, NORAD*
Upregulated in younger trauma patients	*JUNB, PRAM1, NFAM1, CEBPE, CSF3R, ADGRG3, MYO1F, CORO2A, PRKCD, MYH9*
Downregulated in younger trauma patients	*SNORD61, SNORD11, HLA-DRB5, MIR973, SAP30, CRHBP, CAMLG HEMGN, HLF, NBDY*

**Table 3:** Genes with common transcriptomic up or down regulation in HSPCs from older trauma and younger trauma patients (as compared to their age/sex matched healthy controls).

**Expression**	**Genes**
Upregulated	*ABCA7, SLC45A4, ZNF276, CEMP1, TNFAIP2, HCG27, APOBR, METTL7B, BTG2*
Downregulated	*MYCT1, KIT, NRIP1, SPINK2, NPR3, PRKG2, ACSM3, DSG2, MMRN1, FLT3, NOG, EPB41L4A-AS1, ANGPT1, C11orf1, CCDC152, CFH, MEIS1, C3orf80, DPPA4, HNRNPA0, RPS23, NFE2L3, PRKCQ-AS1, PRKCQ, TFPI, AKT3, C1orf21, PAWR, CCDC171, MPP5, PPFIBP1, SFT2D3, ST8SIA6, LOC100130992, ZNF667-AS1, BCL2, CLGN, PLEKHA5, PARP11, ARHGAP5, SLC39A10, SPIN1, WDR35, ZNF711, BBS9, CRISPLD1, DZIP3, FAM135A, FAM213A, HCG4B, LANCL1, ZBTB20, NREP, PDGFC, SCAI*

The older adult trauma cohort was noted to have significant downregulation in the expression of genes involved in hematopoiesis, not seen in younger trauma patients, when compared to age/sex-matched controls (*p* < 0.001). This included, but was not limited to, *CD34, CASP2, CDK6, CXCL12, SMARCA2*, and *SATB1* (Table 4) (37, 41–44). Analysis of genes that were only significant in younger trauma HSPCs (vs. age/sex-matched controls) revealed significant upregulation of genes for receptors important to HSPC proliferation, migration and differentiation, e.g., IL-8Rα, GM-CSFRα, and G-CSFR.

**Table 4:** Prominent genes and miRs found to be significantly altered in old, but not young, bone marrow HSPCs following severe trauma vs. age/sex-matched healthy controls.

**Genes/mi-RNAs**	**Up/down-regulated**	**Function**
*CCR10*	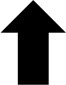	Regulates chemokine expression (*JVI* 2017)
*CCR3*	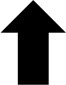	Eosinophil differentiation (*JI* 2003)
*CD34*	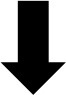	Hematopoietic differentiation (*International Immunology* 1991)
*CXCL12*	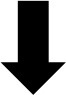	Regulates migration of hematopoietic stem (HSPC) and progenitor cells (*Cytokine* 2015)
miR-125a/b	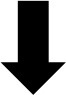	Hematopoietic differentiation and activation of NF-κB (*PNAS* 2012)
*SATB1*	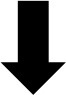	Self-renewal & lymphopoiesis of adult HSPCs (*Cell Reports* 2018)
*TLR5*	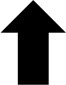	Activation of innate immune response (*Nature* 2001)
*VNN2*	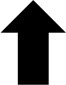	Encodes proteins in hematopoietic cell trafficking (*NCBI* 2019)

**Figure 3:**
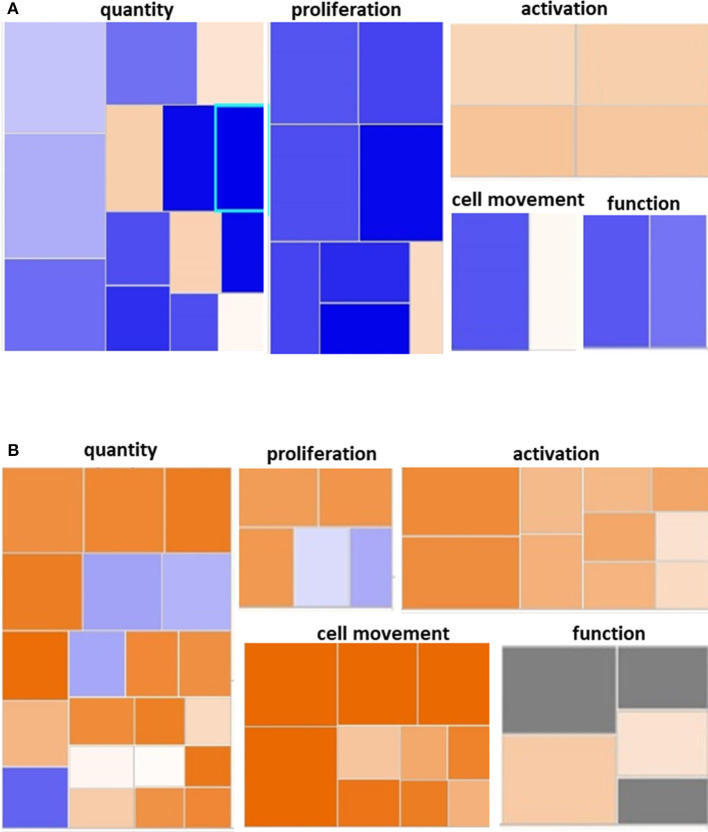
Hematological System Development and Function Pathway from Younger and Older Trauma Patients vs. Age-Matched Controls. Ingenuity Pathway Analysis engendered figure illustrating significant **(A)** down regulation of many genes in the hematological system development and function pathways in older trauma patients as opposed to **(B)** upregulation in younger trauma patients. Orange to red = upregulation, green to blue = downregulation.

**Figure 4:**
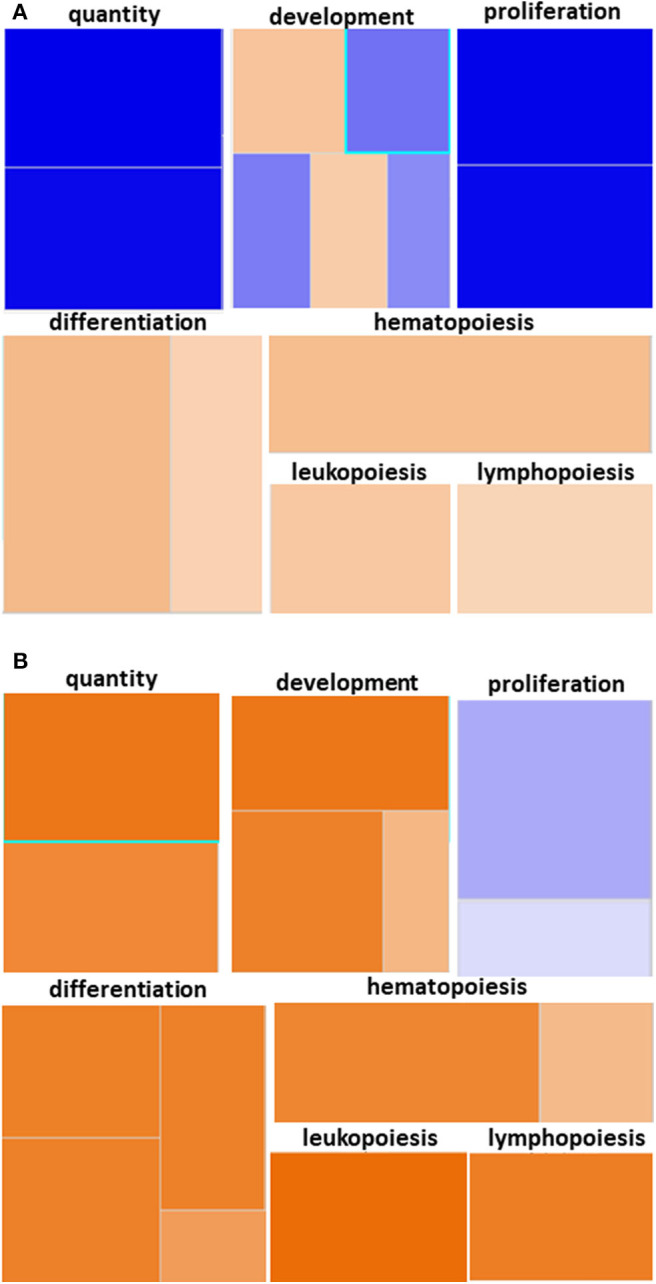
Hematopoiesis Diseases and Functions Pathway from Younger and Older Trauma Patients vs. Age-Matched Controls. Ingenuity Pathway Analysis engendered figure illustrating significant **(A)** down regulation of genes in the hematopoiesis diseases and function pathways in old trauma patients as opposed to **(B)** upregulation in younger trauma patients. Orange to red = upregulation, green to blue = downregulation.

We utilized IPA and GO for further overall and pathway analysis of our genomic data. The use of these software allows greater biological insight into the functional processes activated or inhibited in each trauma group. IPA functional pathways revealed that bone marrow HSPCs from older adult trauma patients had an attenuated transcriptomic/epigenetic response to severe trauma, as displayed in Figure 3. In addition, only HSPCs from younger trauma patients demonstrated significant (z-score > |2|) upregulation of hematopoiesis pathways of function, quantity, and differentiation (Figure 4; Supplemental Tables 1, 2; Supplemental Figure 1).

**Figure 5:**
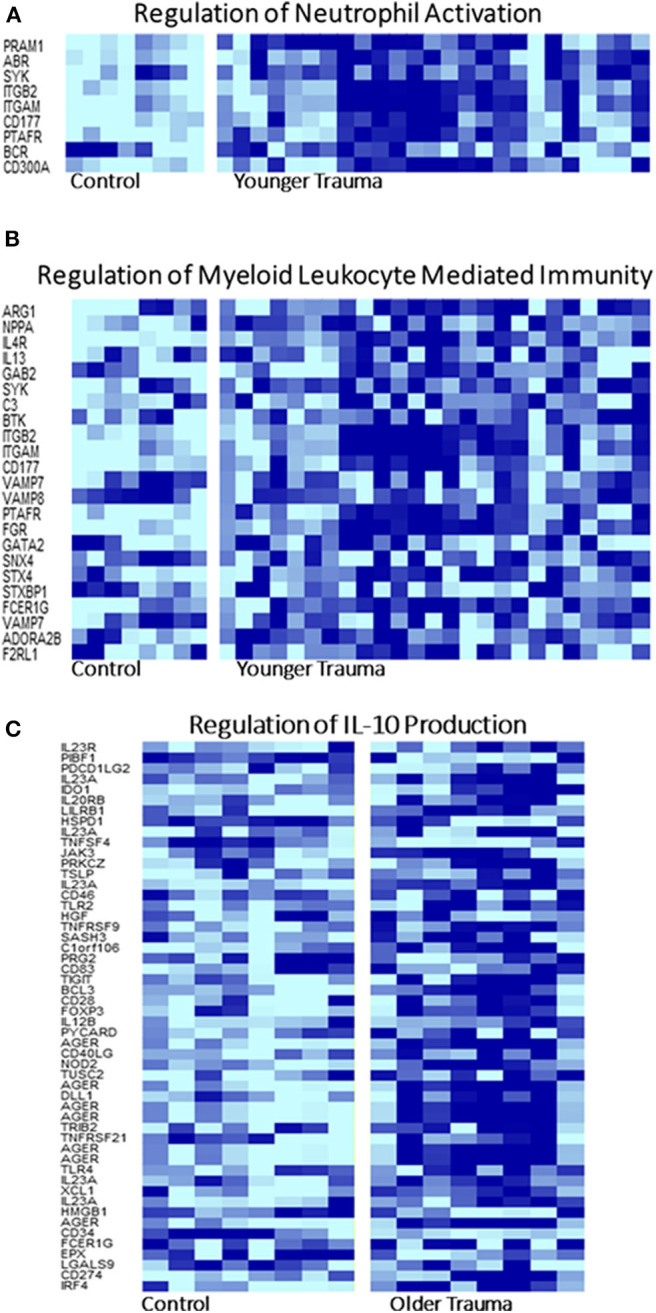
Selected Gene Ontology Pathway Heat Maps in Younger and Older Trauma Patients. Gene ontology pathway analysis demonstrated that several pathways involved in innate and adaptive immunity, **(A)** neutrophil activation and **(B)** myeloid leukocyte mediated immunity, only in the younger trauma patients were significantly different from controls. **(C)** Only older trauma patients had over-representation of the regulation of IL-10 production. Dark blue = upregulation, light blue = down regulation.

GO analysis of the differentially expressed genes illustrated involvement of the older adult HSPC transcriptome mainly in biological processes related to energy and protein metabolism (Supplementary Table 3). Regulation of IL-10 production was only statistically represented in older adult trauma patients, supporting the concept that the inflammatory response of each age group is dissimilar and potentially dysregulated in older vs. younger adults (Figure 5A). The younger trauma patients were predicted to have over-representation of several biological process categories important for immune response, such as myeloid and neutrophil mediated immunity (Figures 5B,C), which was not seen in the older patients (Supplementary Table 4). This post-analysis provided further insights into the important pathways potentially involved in younger and older trauma HSPCs.

### HSPC miR Expression Patterns

A comparison of bone marrow HSPC miR expression from all trauma (*n* = 27) and control (*n* = 16) subjects revealed 60 miRs that were differentially expressed (*p* < 0.005). The expression of 27 miRs were significantly downregulated and 33 were significantly upregulated. Fifteen of these miRs from trauma patients demonstrated at least a 2-fold positive or negative change in expression when compared with control subjects (Table 5). Among these were miRs 125a/b and 146a of which are known to influence HSPC function persistence (45, 46).

**Table 5:** HSPC miRNA from trauma patients with fold-change (FC) > |2|.

**MicroRNA**	**Trauma vs. control FC**
hsa-miR-4454	4.3
hsa-miR-4656	2.2
hsa-miR-193a-5p	4.5
hsa-mir-711	2.1
hsa-miR-185-5p	3.2
hsa-miR-338-5p	2.3
hsa-miR-4284	2.9
snoRNA U62	2.3
hsa-miR-125a-5p	−4.7
hsa-miR-99a-5p	−3.1
hsa-miR-146a-5p	−5.4
hsa-miR-10a-5p	−3
hsa-miR-125b-5p	−5.7
hsa-miR-126-3p	−8
hsa-let-7e-5p	−7.2

Interestingly, miR-125a/b and −146a were amongst the 8 miRs with the highest fold-change difference between expressions of bone marrow HSPC miR from older trauma vs. younger trauma patients. The elderly trauma patients had markedly greater down-regulation of these miRs. Additionally among the miR with the highest fold-change differences, miR-7515 and miR-3128 (47, 48), both implicated in tumor suppression, were upregulated in older trauma patients and down-regulated in younger trauma patients (Table 6).

**Table 6:** miRNA in Human HSPCs from older vs. younger adult trauma patients with largest absolute fold-change (FC) difference.

**miRNA**	**Older adult trauma FC**	**Younger adult trauma FC**	**FC difference**
hsa-miR-3201	−1.8	−10.2	8.4
hsa-miR-150-5p	9.4	1.4	8
hsa-mir-7515	1.8	−5.1	6.9
hsa-miR-3128	1.6	−3.6	5.2
hsa-miR-146a-5p	−9	−2.8	−6.2
hsa-miR-126-3p	−12.7	−6	−6.7
hsa-miR-125a-5p	−9.4	−2.4	−7
hsa-miR-125b-5p	−16.4	−2.4	−14

Analysis using TargetScan revealed that the predicted gene targets of the expressed miR with the largest fold changes from HSPCs isolated from older trauma patients overlap with the genes found significantly expressed in the same subjects (Table 7). The predicted gene targets for the up-regulated miRs had a higher overlap with the genes down-regulated in HSPCs of older trauma patients. These included genes associated with HSPC proliferation and function like *AREG, CXCR1, KIT*, and *MGAM*.

**Table 7:** List of select significant upregulated HSPC miRNAs from old trauma patients and their predicted targets of significant genes of the same cells as analyzed by Target Scan software.

**miRNA**	**Associated genes**
hsa-miR-150-5p	*ACVR1B*
hsa-miR-22-3p	*ACVR1B, PRR14L*
hsa-miR-145-5p	*NR4A2, ACVR1B, ANKFY1, CRK, PRR14L, TSPAN14, ZNF436*
hsa-miR-148a-3p	*KIT*
hsa-mir-7515	*AQP9, CXCR2, GYPE, SART3, SNCA, ANKFY1, MFAP3, MON1B, PRR14L, PTK2B, TRIM27, TSPAN14, ZNF436, ZNF672*
hsa-miR-3128	*MGAM, SNCA, ACVR1B, ANKFY1, C12orf65, MON1B, MYCT1, PAGR1, PRR14L, TNFAIP8L2, TSPAN14, ZKSCAN4, ZNF436*

*Genes in red were significantly down-regulated, while genes in black were significantly upregulated*.

## Discussion

Advanced age is a known risk factor for poor outcomes in trauma patients (11). Increased mortality in the elderly is largely attributed to their diminished physiologic and immunologic reserves, resulting in higher rates of nosocomial infections and sepsis post-trauma (11, 12). Previous murine studies by our laboratory demonstrate that the increased incidence of infection in the elderly is also related to HSPC failure in the aged host after severe injury (18). Therefore, we sought to determine if similar hematopoietic defects are present in humans. While there was no difference in genomic expression patterns based on the magnitude of the traumatic injury (ISS) nor based on blood transfusion in the first 24 h, age was associated with significantly different genomic expression patterns when compared to younger trauma patients of equivalent severity.

Transcriptomic evaluation of bone marrow HSPC from older adult trauma patients illustrates a diminished functional status as well as a blunted capacity for the terminal differentiation of myeloid cells (49). Many genes important in HSPC proliferation, differentiation and mobilization, such as *CD34, CXCL12, miR-125a/b, SATB1, APEX1, and PRDX1*, were all found to be downregulated and only differentially expressed in older trauma HSPCs (50, 51). Interestingly, the genes found differentially expressed only in younger trauma patients were predicted by IPA to have increased activation of hematopoietic function pathways not seen in older trauma patients (Supplemental Tables 1, 2). Pathway analysis also revealed significantly down regulated IL-4 and IL-8 signaling in HSPCs from older trauma patients compared to the younger patients (Supplemental Figure 2).

The older trauma patients show a more blunted and down regulation of many genes in the CXCR4 pathway leading presumably to decreased capacity for cell migration in comparison to the younger trauma patient with significant upregulation in this signaling pathway (Supplemental Figure 3). A few studies have demonstrated that *CXCL12* expressed by surrounding cells in the bone marrow niche promotes HSPC quiescence and retention in the bone marrow via the CXCR4 signaling pathway (52–54). Although, our data demonstrates a downregulation of *CXCL12* in HSPCs of trauma patients in this study, Ding et al. showed that deletion of *CXCL12* in HSPCs had no effect on HSPC mobilization (55). Downregulation of *CXCR4* has been noted in the literature to increase mobilization of HSPCs (56–58). However, we did not find a significant change in CXCR4 gene expression in our study. Additionally, many studies have noted modulation of CXCL12 expression via epigenetics (59–62). Specifically, miR-23a, noted to be upregulated in old trauma patient HSPCs in our study, down-regulates *CXCL12* mRNA and protein expression (59). The role of endogenous CXCL12 is not completely understood, but these points highlight the complexity of the bone marrow niche in the regulation of the HSPC response to certain stressors. More research to study the effect of trauma on specific pathways in the bone marrow niche, its crosstalk with HSPCs, and HSPC migration from the bone marrow is warranted.

Our laboratory previously determined that elderly patients with complicated outcomes have significantly decreased plasma cytokine and chemokine concentrations 0–4 days after severe injury and hemorrhage (11). Interestingly, HSPC genomic analysis in this study revealed many pro-inflammatory related genes differentially expressed and upregulated only in older trauma patients such as TLR5, TLR8, TNFAIP6, TGFA, IL1R2, IL23A, IL7R, CCR3, and IL10RA. However, GO analysis of the differentially expressed genes in the older trauma patients revealed over-representation of biological process categories related to only one inflammatory molecule—IL-10. Additionally, GO revealed over-representation of many processes involved in immune activation in the younger trauma HPSC mRNAs not seen in the older adult patients (Supplementary Tables 3, 4). Taken together, this data suggests that older patients fail to activate the appropriate immune response early after severe injury and show a dysregulated inflammatory response.

miR analysis also revealed differential HSPC regulation after trauma dependent upon age, with significant differences in expression patterns of miRs known to be important for HSPC function. This included increased expression of miR-143/145 in the young, as well as decreased expression of miR-125a/b in the old, all of which are vital to HSPC function (45, 46). Under-expression of miR-143 and−145 have been noted in many malignancies, and overexpression in these malignant cell lines impair cell growth, differentiation, and cause apoptosis of CD34^+^ HSPCs (63, 64). Expression of miR-125a has been noted to increase the number of HSPCs (65), while overexpression of miR-125b in hematopoietic stem cells has been noted to promote self-renewal, differentiation and expansion (66). Additionally, expression of miR-125a/b inhibits TNFAIP3, leading to activation of the NF-κB pathway (45). Evidence indicates that NF-κB signaling leads to a loss of HSPC quiescence and increased differentiation (67). The markedly lowered expression of miR-125a/b seen in HSPCs from the older trauma patients may translate to a markedly decreased NF-κB activity, which in turn contributes to the continued quiescence of HSPCs after traumatic injury.

Our study was limited in several ways. First, HSPCs were negatively isolated by depletion of lineage-committed cells. This is due to the limited amount of bone marrow that can be safely be obtained from these patients, and with hematopoietic stem cells being an extremely rare population. Therefore, the transcriptomics in this study represent all lineage-negative cells including hematopoietic stem and early progenitor cells. HSPCs are known to expand after trauma and also with advanced age, so the expression patterns likely reflect this phenomena as well (18, 49, 68). However, multi-potent progenitor cells (MPPs) and some other progenitor cells, like HSCs, do represent early cell populations present that are host-capable of engendering differentiated hematopoietic cells (69). However, the number of lineage negative cells that we could isolate was limited due to being a rare population, constricting our ability to perform detailed fluorescence-activated cell sorting/flow cytometry. Future studies using novel technology such as single cell RNAseq, Cellular Indexing of Transcriptomes and Epitopes by Sequencing (CITE-Seq), and Assay for Transposase Accessible Chromatin using sequencing (ATAC-Seq) are currently underway in our laboratory and will be required to better comprehend how each cell type may contribute to the suboptimal aged response to injury (70). In addition, sample size and the number of lineage negative cells that we could isolate limited our ability to perform several sub-analyses such as methylcellulose colony assays and functional assays to evaluate mobilization of HSPCs into peripheral blood. Additionally, age-specific data analysis of miR arrays was unable to be performed due to the small sample size of the older adult patient cohort. This may have led to a bias toward the younger adult arrays in the analysis of miR in all trauma patients.

However, trends can still be seen that suggest age-specific differences in epigenetic expression in response to trauma. In addition, we were unable to perform multi-variant logistic regression analysis on all risk factors between older and younger adults (e.g., co-morbidities on arrival) so that age could be determined to be independently associated with the HSPC differences. However, there are limitations to how many bone marrow samples can be obtained in these patients. In addition, in a somewhat practical analysis of older subjects, this is the HSPC response present after trauma for patients ≥55 years old, regardless of other factors (11).

## Conclusions

Bone marrow HSPCs from older human trauma patients have a unique, and in some ways, subdued mRNA/miR response to trauma compared to younger patients. Independent of injury severity and blood transfusion requirement, advanced age may be the key driver of post-traumatic bone marrow HSPC transcriptomic and some epigenetic changes. The regulation of vital miRs and genes involved in HSPC production and differentiation may suggest why older patients have a blunted hematopoietic response to trauma, contributing to their subsequent immune dyscrasia. Although immunomodulation of HSPCs is possible, elderly patients may not respond well to standard cytokines or growth factors. Precision medicine may require epigenetic manipulation to modify HSPC protective immunity to improve long-term outcomes in the elderly.

## Data Availability Statement

The raw data supporting the conclusions of this article will be made available by the authors, without undue reservation, to any qualified researcher.

## Ethics Statement

The studies involving human participants were reviewed and approved by University of Florida Institutional Review Board. The patients/participants provided their written informed consent to participate in this study.

## Author Contributions

Project conception and design: DD, JS, MH, MC, CA, RH, JR, JH, MB, MK, RB, BB, SB, HB, CC, AM, and PE. Data collection: DD, JS, MH, MC, CA, EN, JH, and HP. Data analysis and interpretation: DD, JS, MH, M-CL, ZW, BB, and HB. Original manuscript drafting: DD, MH, and PE. Manuscript review and editing: DD, JS, MH, MC, CA, RH, JR, M-CL, MB, MK, RB, SB, HB, CC, AM, and PE. Manuscript final approval: DD, SB, HB, CC, AM, and PE. All authors contributed to the article and approved the submitted version.

## Conflict of Interest

The authors declare that the research was conducted in the absence of any commercial or financial relationships that could be construed as a potential conflict of interest.
