# Supplementary material for: Identification of Unique mRNA and miRNA Expression Patterns in Bone Marrow Hematopoietic Stem and Progenitor Cells After Trauma in Older Adults
Source: Front Immunol. 2020 Jun 24;11:1289. doi: 10.3389/fimmu.2020.01289 (PMC7326804; doi:10.3389/fimmu.2020.01289)
Supplement: Supplementary file 1 [file Data_Sheet_1.docx]

Supplementary Material

Supplemental Table 1. Hematopoiesis Pathways predicted by IPA to be significantly activated in young trauma patients.

| Diseases or Functions Annotation | Predicted Activation State | Activation z-score | Involved Genes |
| --- | --- | --- | --- |
| Leukopoiesis | Increased | 2.979 | *ADGRG3, ALOX5, ARID3A, BATF, BCL6, C3AR1, CCND3, CEBPE, CFP, CRK, CSF2RA, CSF2RB, CSF3R, CXCR1, CXCR2, DEF6, DGKZ, DUSP10, ELF4, ENTPD1, ERN1, FADD, FCGR1A, FES, HDAC4, IGF2R, IL17RA, IL4R, IL6R, IRF7, ITGAM, ITGB2, JUN, KIT, KLF10, LAT2, LILRA2, MLLT1, MLLT3, MYD88, NFAM1, NLRC4, NR4A2, PAG1, PF4, PIK3AP1, PIK3CD, PIR, PKNOX1, PLCG2, PRKCD, PSTPIP1, PTPN6, RASGRP4, RGL2, RIPK2, RIPK3, SASH3, SEMA4A, SIGLEC10, SLA, SNAI3, SPI1, THEMIS2, TNFRSF1A, TRPM2, TWSG1, TYK2, VAV1, VDR, WAS, ZBTB7A, ZBTB7B* |
| Development of hematopoietic system | Increased | 2.646 | *BCL6, CCND3, CCR1, CSF2RB, CSF3R, CTC1, HOXA10, HOXA5, IL4R, IL6R, KIT, LAT2, MAEA, MRTFA, PF4, PLCG2, PTPN6, RASGRP4, RPS6KA1, SBDS, SPI1, THEMIS2, TNFRSF1A, TYK2, VAV1* |
| Quantity of thymocytes | Increased | 2.616 | *CCND3, CORO1A, CTSD, FADD, GNAI2, ITGB2, KIT, MAML1, PAG1, PIK3CD, SASH3, SNAI3, SPI1, TRAF3IP2, TWSG1, UPF1, VAV1, WAS, ZBTB7B* |
| Lymphopoiesis | Increased | 2.388 | *ADGRG3, ARID3A, BATF, BCL6, C3AR1, CCND3, CSF3R, CXCR1, CXCR2, DEF6,DGKZ,DUSP10,ELF4,ENTPD1,ERN1,FADD,HDAC4,IGF2R,IL17RA,IL4R,IL6R,ITGAM,ITGB2,JUN,KIT,KLF10,LAT2,MYD88,NFAM1,NR4A2,PAG1,PIK3AP1,PIK3CD,PKNOX1,PLCG2,PRKCD,PSTPIP1,PTPN6,RASGRP4,RIPK2,RIPK3,SASH3,SEMA4A,SIGLEC10,SLA,SNAI3,SPI1,THEMIS2,TNFRSF1A,TWSG1,TYK2,VAV1,WAS,ZBTB7A,ZBTB7B* |
| Differentiation of myeloid leukocytes | Increased | 2.31 | *ALOX5,CEBPE,CFP,CRK,CSF2RA,CSF2RB,CSF3R,CXCR2,FADD,FES,IL17RA,IRF7,ITGAM,JUN,KIT,LILRA2,MLLT1,MLLT3,MYD88,NLRC4,PF4,PIK3CD,PIR,PTPN6,RGL2,SPI1,VDR* |
| T cell development | Increased | 2.294 | *BATF,BCL6,C3AR1,CXCR1,CXCR2,DEF6,DUSP10,ELF4,ENTPD1,FADD,IGF2R,IL17RA,IL4R,IL6R,ITGB2,JUN,KIT,KLF10,LAT2,MYD88,NR4A2,PAG1,PIK3CD,PKNOX1,PTPN6,RASGRP4,RIPK2,RIPK3,SASH3,SEMA4A,SLA,SNAI3,SPI1,THEMIS2,TNFRSF1A,TWSG1,TYK2,VAV1,WAS,ZBTB7B* |
| Differentiation of phagocytes | Increased | 2.287 | *ALOX5,BATF,BCL6,CEBPE,CRK,CSF2RA,CSF2RB,CSF3R,ERN1,FADD,FCGR1A,FES,IRF7,ITGAM,JUN,KIT,LILRA2,MLLT1,MLLT3,MYD88,NLRC4,PF4,PIK3CD,PIR,PTPN6,RGL2,SLA,SPI1,TNFRSF1A,TRPM2,VDR* |
| Differentiation of macrophages | Increased | 2.284 | *ALOX5,CEBPE,CRK,CSF2RA,CSF2RB,FADD,FES,IRF7,ITGAM,MLLT1,MLLT3,MYD88,NLRC4,PF4,SPI1,VDR* |
| Differentiation of antigen presenting cells | Increased | 2.28 | *ALOX5,BATF,BCL6,CEBPE,CRK,CSF2RA,CSF2RB,ERN1,FADD,FCGR1A,FES,IRF7,ITGAM,LILRA2,MLLT1,MLLT3,MYD88,NLRC4,PF4,SLA,SPI1,TNFRSF1A,TRPM2,VDR* |
| Hematopoiesis of mononuclear leukocytes | Increased | 2.169 | *ADGRG3,ARID3A,BATF,BCL6,C3AR1,CCND3,CSF2RB,CSF3R,CXCR1,CXCR2,DEF6,DGKZ,DUSP10,ELF4,ENTPD1,ERN1,FADD,HDAC4,IGF2R,IL17RA,IL4R,IL6R,IRF7,ITGAM,ITGB2,JUN,KIT,KLF10,LAT2,LILRA2,MYD88,NFAM1,NR4A2,PAG1,PF4,PIK3AP1,PIK3CD,PIR,PKNOX1,PLCG2,PRKCD,PSTPIP1,PTPN6,RASGRP4,RIPK2,RIPK3,SASH3,SEMA4A,SIGLEC10,SLA,SNAI3,SPI1,THEMIS2,TNFRSF1A,TWSG1,TYK2,VAV1,WAS,ZBTB7A,ZBTB7B* |
| Quantity of hematopoietic progenitor cells | Increased | 2.043 | *ADGRG3,ARID3A,CCND3,CORO1A,CSF3R,CTC1,CTSD,ELF4,FADD,FES,GNAI2,HOXA5,ITGB2,KIT,MAML1,MYD88,NBEAL2,PAG1,PIK3AP1,PIK3CD,PLCG2,PRKCD,PTPN6,RASSF2,SASH3,SH3BP2,SIRPA,SNAI3,SPI1,TCIRG1,TRAF3IP2,TWSG1,UPF1,VASP,VAV1,WAS,ZBTB7A,ZBTB7B* |

Supplemental Table 2. Hematopoiesis Pathways predicted by IPA to be significantly activated in older trauma patients.

| Diseases or Functions Annotation | Predicted Activation State | Activation z-score | Involved Genes |
| --- | --- | --- | --- |
| Quantity of pre-B lymphocytes | Decreased | -2.542 | *BCL2, CXCL12, FLI1, FLT3, FNIP1, HOXA9, IKZF3, IL7R, KLF2, MS4A1, MYB, PIK3R1, SOX4, TCF12, TCF4, TEC* |
| Proliferation of pro-B lymphocytes | Decreased | -2.423 | *BCL11A, BCL2, CXCL12, FLT3, LILRB4, MYB* |
| Proliferation of hematopoietic progenitor cells | Decreased | -2.347 | *BCL11A, BCL2, CBFB, CCL3, CD2, CD34, CD3E, CXCL12, ERG, ETV6, FLI1, FLT3, FUBP1, GRAP2, HOXA9, IL7R, KIT, KLF2, LILRB4, MEIS1,* mir-486*, MLLT3, MYB, NF1, NFATC3, PIK3R1, PRDM1, SMAD5, TAC4, TCF12, TOX* |
| Quantity of hematopoietic progenitor cells | Decreased | -2.339 | *BCL11A, BCL2, CBFB, CD247, CD34, CD3E, CD3G, CDK6, CRIP3, CXCL12, FCGR2B, FLI1, FLT3, FNIP1, FUBP1, GRAP2, HOXA9, IKZF3, IL7R, KIT, KLF2, LILRB3, MBTD1, MIR17HG, MS4A1, MYB, NOG, PIK3R1, PRDX1, PRKCQ, RPS6, SATB1, SMAD5, SOX4, TAC4, TCF12, TCF4, TEC, TOX, TPP2, TSC22D3* |

Supplemental Table 3. Summary of Gene Ontology gene sets found to be significantly represented by older trauma HSPC mRNA. 24 Biological Process (BP) categories and 1 Molecular Function (MF) categories are significant.

| GO category | GO ontology | GO term | Number of genes involved | LS permutation p-value | KS permutation p-value | Efron-Tibshirani's GSA test p-value |
| --- | --- | --- | --- | --- | --- | --- |
| GO:0032613 | BP | interleukin-10 production | [54](file:///C:\Users\d.darden\AppData\Old%20Trauma%20vs%20CTL%20GO\GeneSetGenesTable1.html#GO:0032613) | 0.00001 | 0.00458 | < 0.005 (+) |
| GO:0032653 | BP | regulation of interleukin-10 production | [52](file:///C:\Users\d.darden\AppData\Old%20Trauma%20vs%20CTL%20GO\GeneSetGenesTable1.html#GO:0032653) | 0.00001 | 0.00186 | < 0.005 (+) |
| GO:0032693 | BP | negative regulation of interleukin-10 production | [26](file:///C:\Users\d.darden\AppData\Old%20Trauma%20vs%20CTL%20GO\GeneSetGenesTable1.html#GO:0032693) | 0.00001 | 0.00413 | < 0.005 (+) |
| GO:0043030 | BP | regulation of macrophage activation | [42](file:///C:\Users\d.darden\AppData\Old%20Trauma%20vs%20CTL%20GO\GeneSetGenesTable1.html#GO:0043030) | 0.00001 | 0.00177 | 0.005 (+) |
| GO:0043032 | BP | positive regulation of macrophage activation | [28](file:///C:\Users\d.darden\AppData\Old%20Trauma%20vs%20CTL%20GO\GeneSetGenesTable1.html#GO:0043032) | 0.00001 | 0.00001 | < 0.005 (+) |
| GO:0106014 | BP | regulation of inflammatory response to wounding | [9](file:///C:\Users\d.darden\AppData\Old%20Trauma%20vs%20CTL%20GO\GeneSetGenesTable1.html#GO:0106014) | 0.00001 | 0.00001 | < 0.005 (+) |
| GO:0106015 | BP | negative regulation of inflammatory response to wounding | [8](file:///C:\Users\d.darden\AppData\Old%20Trauma%20vs%20CTL%20GO\GeneSetGenesTable1.html#GO:0106015) | 0.00001 | 0.00001 | < 0.005 (+) |
| GO:1904596 | BP | regulation of connective tissue replacement involved in inflammatory response wound healing | [7](file:///C:\Users\d.darden\AppData\Old%20Trauma%20vs%20CTL%20GO\GeneSetGenesTable1.html#GO:1904596) | 0.00001 | 0.00001 | < 0.005 (+) |
| GO:1905204 | BP | negative regulation of connective tissue replacement | [7](file:///C:\Users\d.darden\AppData\Old%20Trauma%20vs%20CTL%20GO\GeneSetGenesTable1.html#GO:1905204) | 0.00001 | 0.00001 | < 0.005 (+) |
| GO:0030449 | BP | regulation of complement activation | [57](file:///C:\Users\d.darden\AppData\Old%20Trauma%20vs%20CTL%20GO\GeneSetGenesTable3.html#GO:0030449) | 0.00259 | 0.00016 | 0.005 (+) |
| GO:0009756 | BP | carbohydrate mediated signaling | [13](file:///C:\Users\d.darden\AppData\Old%20Trauma%20vs%20CTL%20GO\GeneSetGenesTable1.html#GO:0009756) | 0.00001 | 0.00459 | < 0.005 (+) |
| GO:0009757 | BP | hexose mediated signaling | [11](file:///C:\Users\d.darden\AppData\Old%20Trauma%20vs%20CTL%20GO\GeneSetGenesTable1.html#GO:0009757) | 0.00001 | 0.00148 | < 0.005 (+) |
| GO:0010182 | BP | sugar mediated signaling pathway | [11](file:///C:\Users\d.darden\AppData\Old%20Trauma%20vs%20CTL%20GO\GeneSetGenesTable1.html#GO:0010182) | 0.00001 | 0.00148 | < 0.005 (+) |
| GO:0010255 | BP | glucose mediated signaling pathway | [11](file:///C:\Users\d.darden\AppData\Old%20Trauma%20vs%20CTL%20GO\GeneSetGenesTable1.html#GO:0010255) | 0.00001 | 0.00148 | < 0.005 (+) |
| GO:0014002 | BP | astrocyte development | [37](file:///C:\Users\d.darden\AppData\Old%20Trauma%20vs%20CTL%20GO\GeneSetGenesTable1.html#GO:0014002) | 0.00001 | 0.00189 | < 0.005 (+) |
| GO:0019081 | BP | viral translation | [12](file:///C:\Users\d.darden\AppData\Old%20Trauma%20vs%20CTL%20GO\GeneSetGenesTable1.html#GO:0019081) | 0.00001 | 0.00017 | 0.005 (-) |
| GO:0048143 | BP | astrocyte activation | [18](file:///C:\Users\d.darden\AppData\Old%20Trauma%20vs%20CTL%20GO\GeneSetGenesTable1.html#GO:0048143) | 0.00001 | 0.00033 | < 0.005 (+) |
| GO:0061888 | BP | regulation of astrocyte activation | [10](file:///C:\Users\d.darden\AppData\Old%20Trauma%20vs%20CTL%20GO\GeneSetGenesTable1.html#GO:0061888) | 0.00001 | 0.00029 | < 0.005 (+) |
| GO:1900744 | BP | regulation of p38MAPK cascade | [45](file:///C:\Users\d.darden\AppData\Old%20Trauma%20vs%20CTL%20GO\GeneSetGenesTable1.html#GO:1900744) | 0.00001 | 0.0042 | < 0.005 (+) |
| GO:1904603 | BP | regulation of advanced glycation end-product receptor activity | [7](file:///C:\Users\d.darden\AppData\Old%20Trauma%20vs%20CTL%20GO\GeneSetGenesTable1.html#GO:1904603) | 0.00001 | 0.00001 | < 0.005 (+) |
| GO:0048708 | BP | astrocyte differentiation | [75](file:///C:\Users\d.darden\AppData\Old%20Trauma%20vs%20CTL%20GO\GeneSetGenesTable1.html#GO:0048708) | 0.00002 | 0.0008 | < 0.005 (+) |
| GO:0010603 | BP | regulation of cytoplasmic mRNA processing body assembly | [9](file:///C:\Users\d.darden\AppData\Old%20Trauma%20vs%20CTL%20GO\GeneSetGenesTable1.html#GO:0010603) | 0.00008 | 0.00003 | 0.005 (-) |
| GO:0001083 | MF | transcription factor activity, RNA polymerase II basal transcription factor binding | [6](file:///C:\Users\d.darden\AppData\Old%20Trauma%20vs%20CTL%20GO\GeneSetGenesTable2.html#GO:0001083) | 0.00035 | 0.00058 | < 0.005 (-) |
| GO:2000257 | BP | regulation of protein activation cascade | [58](file:///C:\Users\d.darden\AppData\Old%20Trauma%20vs%20CTL%20GO\GeneSetGenesTable3.html#GO:2000257) | 0.00393 | 0.00035 | 0.005 (+) |

Supplemental Table 4. Summary of Gene Ontology gene sets found to be significantly represented by young trauma HSPC mRNA. 57 Biological Process (BP) categories and 5 Cellular Component (CC) categories are significant.

| GO category | GO ontology | GO term | Number of genes | LS permutation p-value | KS permutation p-value | Efron-Tibshirani's GSA test p-value |
| --- | --- | --- | --- | --- | --- | --- |
| GO:0002886 | BP | regulation of myeloid leukocyte mediated immunity | 45 | 0.00001 | 0.00003 | < 0.005 (+) |
| GO:0032728 | BP | positive regulation of interferon-beta production | 35 | 0.00001 | 0.00001 | < 0.005 (+) |
| GO:0033003 | BP | regulation of mast cell activation | [41](file:///C:\Users\d.darden\AppData\Young%20Trauma%20vs%20CTL%20GO%20p005\GeneSetGenesTable1.html#GO:0033003) | 0.00001 | 0.00277 | < 0.005 (+) |
| GO:0033006 | BP | regulation of mast cell activation involved in immune response | [30](file:///C:\Users\d.darden\AppData\Young%20Trauma%20vs%20CTL%20GO%20p005\GeneSetGenesTable1.html#GO:0033006) | 0.00001 | 0.00286 | < 0.005 (+) |
| GO:0034121 | BP | regulation of toll-like receptor signaling pathway | [68](file:///C:\Users\d.darden\AppData\Young%20Trauma%20vs%20CTL%20GO%20p005\GeneSetGenesTable1.html#GO:0034121) | 0.00001 | 0.00001 | < 0.005 (+) |
| GO:0034138 | BP | toll-like receptor 3 signaling pathway | [26](file:///C:\Users\d.darden\AppData\Young%20Trauma%20vs%20CTL%20GO%20p005\GeneSetGenesTable1.html#GO:0034138) | 0.00001 | 0.00001 | 0.005 (+) |
| GO:0034139 | BP | regulation of toll-like receptor 3 signaling pathway | [16](file:///C:\Users\d.darden\AppData\Young%20Trauma%20vs%20CTL%20GO%20p005\GeneSetGenesTable1.html#GO:0034139) | 0.00001 | 0.00043 | 0.005 (+) |
| GO:0043300 | BP | regulation of leukocyte degranulation | [43](file:///C:\Users\d.darden\AppData\Young%20Trauma%20vs%20CTL%20GO%20p005\GeneSetGenesTable1.html#GO:0043300) | 0.00001 | 0.00008 | 0.005 (+) |
| GO:0043304 | BP | regulation of mast cell degranulation | [30](file:///C:\Users\d.darden\AppData\Young%20Trauma%20vs%20CTL%20GO%20p005\GeneSetGenesTable1.html#GO:0043304) | 0.00001 | 0.00286 | < 0.005 (+) |
| GO:0043371 | BP | negative regulation of CD4-positive, alpha-beta T cell differentiation | [15](file:///C:\Users\d.darden\AppData\Young%20Trauma%20vs%20CTL%20GO%20p005\GeneSetGenesTable1.html#GO:0043371) | 0.00001 | 0.00476 | < 0.005 (+) |
| GO:0045063 | BP | T-helper 1 cell differentiation | [17](file:///C:\Users\d.darden\AppData\Young%20Trauma%20vs%20CTL%20GO%20p005\GeneSetGenesTable1.html#GO:0045063) | 0.00001 | 0.00425 | < 0.005 (+) |
| GO:0045576 | BP | mast cell activation | [58](file:///C:\Users\d.darden\AppData\Young%20Trauma%20vs%20CTL%20GO%20p005\GeneSetGenesTable1.html#GO:0045576) | 0.00001 | 0.00149 | 0.005 (+) |
| GO:0046636 | BP | negative regulation of alpha-beta T cell activation | [28](file:///C:\Users\d.darden\AppData\Young%20Trauma%20vs%20CTL%20GO%20p005\GeneSetGenesTable1.html#GO:0046636) | 0.00001 | 0.00392 | < 0.005 (+) |
| GO:0050710 | BP | negative regulation of cytokine secretion | [77](file:///C:\Users\d.darden\AppData\Young%20Trauma%20vs%20CTL%20GO%20p005\GeneSetGenesTable1.html#GO:0050710) | 0.00001 | 0.00001 | 0.005 (+) |
| GO:2000515 | BP | negative regulation of CD4-positive, alpha-beta T cell activation | [20](file:///C:\Users\d.darden\AppData\Young%20Trauma%20vs%20CTL%20GO%20p005\GeneSetGenesTable1.html#GO:2000515) | 0.00001 | 0.00178 | < 0.005 (+) |
| GO:0032720 | BP | negative regulation of tumor necrosis factor production | [56](file:///C:\Users\d.darden\AppData\Young%20Trauma%20vs%20CTL%20GO%20p005\GeneSetGenesTable2.html#GO:0032720) | 0.00005 | 0.00001 | < 0.005 (+) |
| GO:1903556 | BP | negative regulation of tumor necrosis factor superfamily cytokine production | [58](file:///C:\Users\d.darden\AppData\Young%20Trauma%20vs%20CTL%20GO%20p005\GeneSetGenesTable2.html#GO:1903556) | 0.00006 | 0.00001 | < 0.005 (+) |
| GO:1902563 | BP | regulation of neutrophil activation | [9](file:///C:\Users\d.darden\AppData\Young%20Trauma%20vs%20CTL%20GO%20p005\GeneSetGenesTable2.html#GO:1902563) | 0.00007 | 0.00045 | 0.005 (+) |
| GO:0043313 | BP | regulation of neutrophil degranulation | [8](file:///C:\Users\d.darden\AppData\Young%20Trauma%20vs%20CTL%20GO%20p005\GeneSetGenesTable2.html#GO:0043313) | 0.00013 | 0.00188 | 0.005 (+) |
| GO:0002888 | BP | positive regulation of myeloid leukocyte mediated immunity | [23](file:///C:\Users\d.darden\AppData\Young%20Trauma%20vs%20CTL%20GO%20p005\GeneSetGenesTable3.html#GO:0002888) | 0.0002 | 0.00197 | < 0.005 (+) |
| GO:0034162 | BP | toll-like receptor 9 signaling pathway | [18](file:///C:\Users\d.darden\AppData\Young%20Trauma%20vs%20CTL%20GO%20p005\GeneSetGenesTable3.html#GO:0034162) | 0.00034 | 0.00119 | 0.005 (+) |
| GO:0070673 | BP | response to interleukin-18 | [10](file:///C:\Users\d.darden\AppData\Young%20Trauma%20vs%20CTL%20GO%20p005\GeneSetGenesTable3.html#GO:0070673) | 0.00048 | 0.00475 | < 0.005 (+) |
| GO:0034122 | BP | negative regulation of toll-like receptor signaling pathway | [37](file:///C:\Users\d.darden\AppData\Young%20Trauma%20vs%20CTL%20GO%20p005\GeneSetGenesTable4.html#GO:0034122) | 0.00097 | 0.00001 | 0.005 (+) |
| GO:0001562 | BP | response to protozoan | [19](file:///C:\Users\d.darden\AppData\Young%20Trauma%20vs%20CTL%20GO%20p005\GeneSetGenesTable1.html#GO:0001562) | 0.00001 | 0.00003 | < 0.005 (+) |
| GO:0002090 | BP | regulation of receptor internalization | [44](file:///C:\Users\d.darden\AppData\Young%20Trauma%20vs%20CTL%20GO%20p005\GeneSetGenesTable1.html#GO:0002090) | 0.00001 | 0.00396 | < 0.005 (+) |
| GO:0002181 | BP | cytoplasmic translation | [75](file:///C:\Users\d.darden\AppData\Young%20Trauma%20vs%20CTL%20GO%20p005\GeneSetGenesTable1.html#GO:0002181) | 0.00001 | 0.00001 | < 0.005 (-) |
| GO:0002820 | BP | negative regulation of adaptive immune response | [43](file:///C:\Users\d.darden\AppData\Young%20Trauma%20vs%20CTL%20GO%20p005\GeneSetGenesTable1.html#GO:0002820) | 0.00001 | 0.00042 | < 0.005 (+) |
| GO:0006678 | BP | glucosylceramide metabolic process | [7](file:///C:\Users\d.darden\AppData\Young%20Trauma%20vs%20CTL%20GO%20p005\GeneSetGenesTable1.html#GO:0006678) | 0.00001 | 0.00097 | < 0.005 (+) |
| GO:0007009 | BP | plasma membrane organization | [83](file:///C:\Users\d.darden\AppData\Young%20Trauma%20vs%20CTL%20GO%20p005\GeneSetGenesTable1.html#GO:0007009) | 0.00001 | 0.00006 | 0.005 (+) |
| GO:0010664 | BP | negative regulation of striated muscle cell apoptotic process | [38](file:///C:\Users\d.darden\AppData\Young%20Trauma%20vs%20CTL%20GO%20p005\GeneSetGenesTable1.html#GO:0010664) | 0.00001 | 0.00444 | 0.005 (+) |
| GO:0031532 | BP | actin cytoskeleton reorganization | [99](file:///C:\Users\d.darden\AppData\Young%20Trauma%20vs%20CTL%20GO%20p005\GeneSetGenesTable1.html#GO:0031532) | 0.00001 | 0.00043 | 0.005 (+) |
| GO:0031623 | BP | receptor internalization | [97](file:///C:\Users\d.darden\AppData\Young%20Trauma%20vs%20CTL%20GO%20p005\GeneSetGenesTable1.html#GO:0031623) | 0.00001 | 0.00001 | 0.005 (+) |
| GO:0032225 | BP | regulation of synaptic transmission, dopaminergic | [23](file:///C:\Users\d.darden\AppData\Young%20Trauma%20vs%20CTL%20GO%20p005\GeneSetGenesTable1.html#GO:0032225) | 0.00001 | 0.00079 | < 0.005 (+) |
| GO:0032226 | BP | positive regulation of synaptic transmission, dopaminergic | [14](file:///C:\Users\d.darden\AppData\Young%20Trauma%20vs%20CTL%20GO%20p005\GeneSetGenesTable1.html#GO:0032226) | 0.00001 | 0.0002 | < 0.005 (+) |
| GO:0032484 | BP | Ral protein signal transduction | [7](file:///C:\Users\d.darden\AppData\Young%20Trauma%20vs%20CTL%20GO%20p005\GeneSetGenesTable1.html#GO:0032484) | 0.00001 | 0.00041 | < 0.005 (+) |
| GO:0032897 | BP | negative regulation of viral transcription | [36](file:///C:\Users\d.darden\AppData\Young%20Trauma%20vs%20CTL%20GO%20p005\GeneSetGenesTable1.html#GO:0032897) | 0.00001 | 0.00061 | < 0.005 (+) |
| GO:0042073 | BP | intraciliary transport | [49](file:///C:\Users\d.darden\AppData\Young%20Trauma%20vs%20CTL%20GO%20p005\GeneSetGenesTable1.html#GO:0042073) | 0.00001 | 0.00001 | 0.005 (-) |
| GO:0044854 | BP | plasma membrane raft assembly | [10](file:///C:\Users\d.darden\AppData\Young%20Trauma%20vs%20CTL%20GO%20p005\GeneSetGenesTable1.html#GO:0044854) | 0.00001 | 0.00019 | < 0.005 (+) |
| GO:0044857 | BP | plasma membrane raft organization | [10](file:///C:\Users\d.darden\AppData\Young%20Trauma%20vs%20CTL%20GO%20p005\GeneSetGenesTable1.html#GO:0044857) | 0.00001 | 0.00019 | < 0.005 (+) |
| GO:0060352 | BP | cell adhesion molecule production | [16](file:///C:\Users\d.darden\AppData\Young%20Trauma%20vs%20CTL%20GO%20p005\GeneSetGenesTable1.html#GO:0060352) | 0.00001 | 0.00031 | < 0.005 (+) |
| GO:0060353 | BP | regulation of cell adhesion molecule production | [14](file:///C:\Users\d.darden\AppData\Young%20Trauma%20vs%20CTL%20GO%20p005\GeneSetGenesTable1.html#GO:0060353) | 0.00001 | 0.0002 | < 0.005 (+) |
| GO:1901739 | BP | regulation of myoblast fusion | [27](file:///C:\Users\d.darden\AppData\Young%20Trauma%20vs%20CTL%20GO%20p005\GeneSetGenesTable1.html#GO:1901739) | 0.00001 | 0.00088 | 0.005 (+) |
| GO:1903044 | BP | protein localization to membrane raft | [16](file:///C:\Users\d.darden\AppData\Young%20Trauma%20vs%20CTL%20GO%20p005\GeneSetGenesTable1.html#GO:1903044) | 0.00001 | 0.00063 | 0.005 (+) |
| GO:0002691 | BP | regulation of cellular extravasation | [23](file:///C:\Users\d.darden\AppData\Young%20Trauma%20vs%20CTL%20GO%20p005\GeneSetGenesTable2.html#GO:0002691) | 0.00002 | 0.00001 | < 0.005 (+) |
| GO:2001244 | BP | positive regulation of intrinsic apoptotic signaling pathway | [52](file:///C:\Users\d.darden\AppData\Young%20Trauma%20vs%20CTL%20GO%20p005\GeneSetGenesTable2.html#GO:2001244) | 0.00007 | 0.00001 | 0.005 (-) |
| GO:0060544 | BP | regulation of necroptotic process | [18](file:///C:\Users\d.darden\AppData\Young%20Trauma%20vs%20CTL%20GO%20p005\GeneSetGenesTable2.html#GO:0060544) | 0.00014 | 0.00118 | < 0.005 (+) |
| GO:0070206 | BP | protein trimerization | [53](file:///C:\Users\d.darden\AppData\Young%20Trauma%20vs%20CTL%20GO%20p005\GeneSetGenesTable3.html#GO:0070206) | 0.00021 | 0.00074 | 0.005 (+) |
| GO:0031063 | BP | regulation of histone deacetylation | [26](file:///C:\Users\d.darden\AppData\Young%20Trauma%20vs%20CTL%20GO%20p005\GeneSetGenesTable3.html#GO:0031063) | 0.00034 | 0.00402 | < 0.005 (+) |
| GO:0006054 | BP | N-acetylneuraminate metabolic process | [11](file:///C:\Users\d.darden\AppData\Young%20Trauma%20vs%20CTL%20GO%20p005\GeneSetGenesTable3.html#GO:0006054) | 0.00035 | 0.00003 | 0.005 (+) |
| GO:0031065 | BP | positive regulation of histone deacetylation | [17](file:///C:\Users\d.darden\AppData\Young%20Trauma%20vs%20CTL%20GO%20p005\GeneSetGenesTable3.html#GO:0031065) | 0.00063 | 0.00154 | < 0.005 (+) |
| GO:0009083 | BP | branched-chain amino acid catabolic process | [21](file:///C:\Users\d.darden\AppData\Young%20Trauma%20vs%20CTL%20GO%20p005\GeneSetGenesTable4.html#GO:0009083) | 0.00094 | 0.00112 | 0.005 (-) |
| GO:0042451 | BP | purine nucleoside biosynthetic process | [21](file:///C:\Users\d.darden\AppData\Young%20Trauma%20vs%20CTL%20GO%20p005\GeneSetGenesTable4.html#GO:0042451) | 0.00142 | 0.0028 | < 0.005 (-) |
| GO:0046129 | BP | purine ribonucleoside biosynthetic process | [21](file:///C:\Users\d.darden\AppData\Young%20Trauma%20vs%20CTL%20GO%20p005\GeneSetGenesTable4.html#GO:0046129) | 0.00142 | 0.0028 | < 0.005 (-) |
| GO:0042455 | BP | ribonucleoside biosynthetic process | [37](file:///C:\Users\d.darden\AppData\Young%20Trauma%20vs%20CTL%20GO%20p005\GeneSetGenesTable4.html#GO:0042455) | 0.00161 | 0.00029 | 0.005 (-) |
| GO:0009081 | BP | branched-chain amino acid metabolic process | [24](file:///C:\Users\d.darden\AppData\Young%20Trauma%20vs%20CTL%20GO%20p005\GeneSetGenesTable5.html#GO:0009081) | 0.00171 | 0.00477 | 0.005 (-) |
| GO:0006627 | BP | protein processing involved in protein targeting to mitochondrion | [8](file:///C:\Users\d.darden\AppData\Young%20Trauma%20vs%20CTL%20GO%20p005\GeneSetGenesTable5.html#GO:0006627) | 0.00184 | 0.00196 | 0.005 (-) |
| GO:0018206 | BP | peptidyl-methionine modification | [11](file:///C:\Users\d.darden\AppData\Young%20Trauma%20vs%20CTL%20GO%20p005\GeneSetGenesTable5.html#GO:0018206) | 0.00269 | 0.00022 | 0.005 (-) |
| GO:0001931 | CC | uropod | [20](file:///C:\Users\d.darden\AppData\Young%20Trauma%20vs%20CTL%20GO%20p005\GeneSetGenesTable1.html#GO:0001931) | 0.00001 | 0.00001 | < 0.005 (+) |
| GO:0030864 | CC | cortical actin cytoskeleton | [77](file:///C:\Users\d.darden\AppData\Young%20Trauma%20vs%20CTL%20GO%20p005\GeneSetGenesTable1.html#GO:0030864) | 0.00001 | 0.00208 | < 0.005 (+) |
| GO:0030990 | CC | intraciliary transport particle | [30](file:///C:\Users\d.darden\AppData\Young%20Trauma%20vs%20CTL%20GO%20p005\GeneSetGenesTable1.html#GO:0030990) | 0.00001 | 0.00001 | 0.005 (-) |
| GO:0031254 | CC | cell trailing edge | [20](file:///C:\Users\d.darden\AppData\Young%20Trauma%20vs%20CTL%20GO%20p005\GeneSetGenesTable2.html#GO:0031254) | 0.00001 | 0.00001 | < 0.005 (+) |
| GO:0035869 | CC | ciliary transition zone | [63](file:///C:\Users\d.darden\AppData\Young%20Trauma%20vs%20CTL%20GO%20p005\GeneSetGenesTable2.html#GO:0035869) | 0.00002 | 0.00025 | 0.005 (-) |

**A
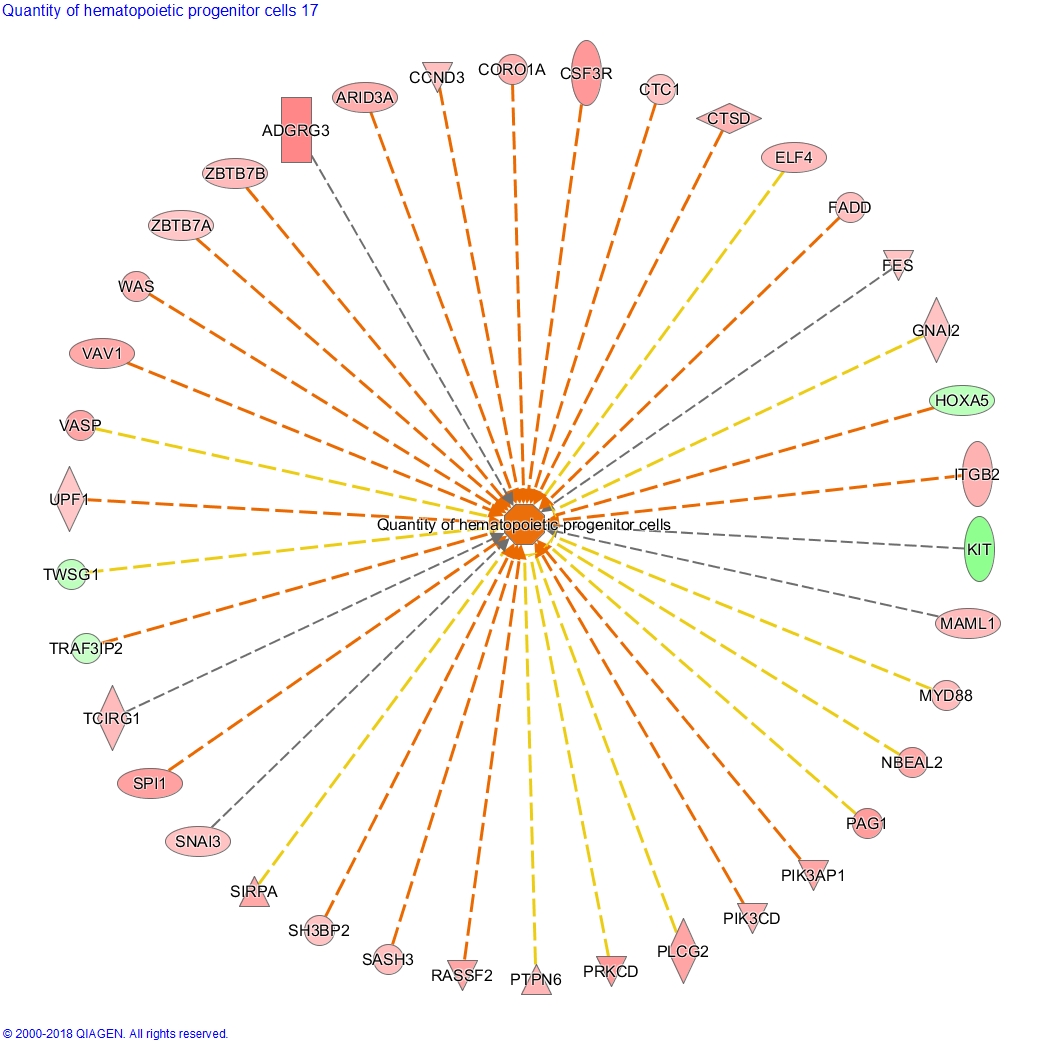
**

**B**

**
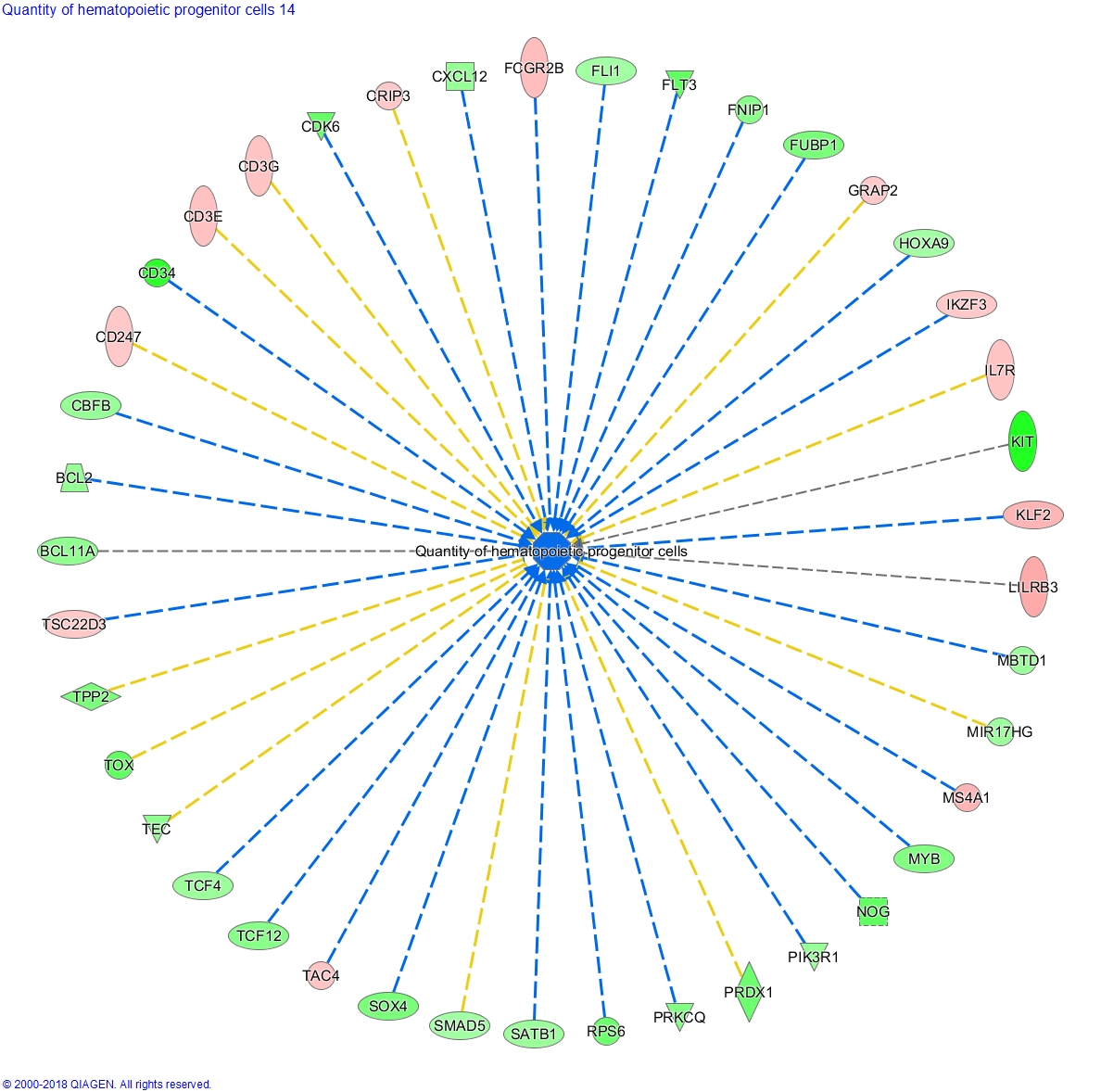
**

**Supplementary Figure 1.** IPA illustration showing regulation of differentially expressed genes important for the quantity of hematopoietic progenitor cells in (**A**) young and (**B**) old adult trauma patients. Orange to red = upregulation, green to blue = downregulation.

| **A**  Extracellular space  Cytoplasm | 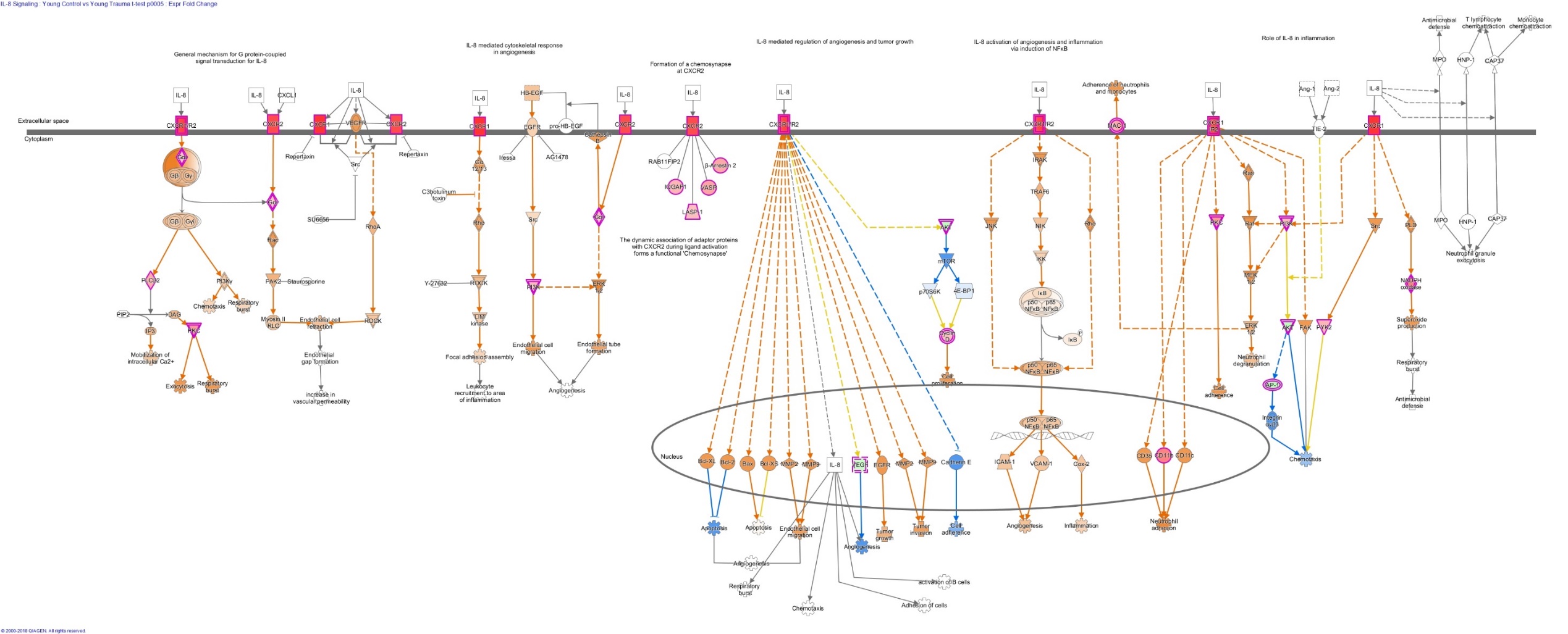 |
| --- | --- |
| **B**  Extracellular space  Cytoplasm | 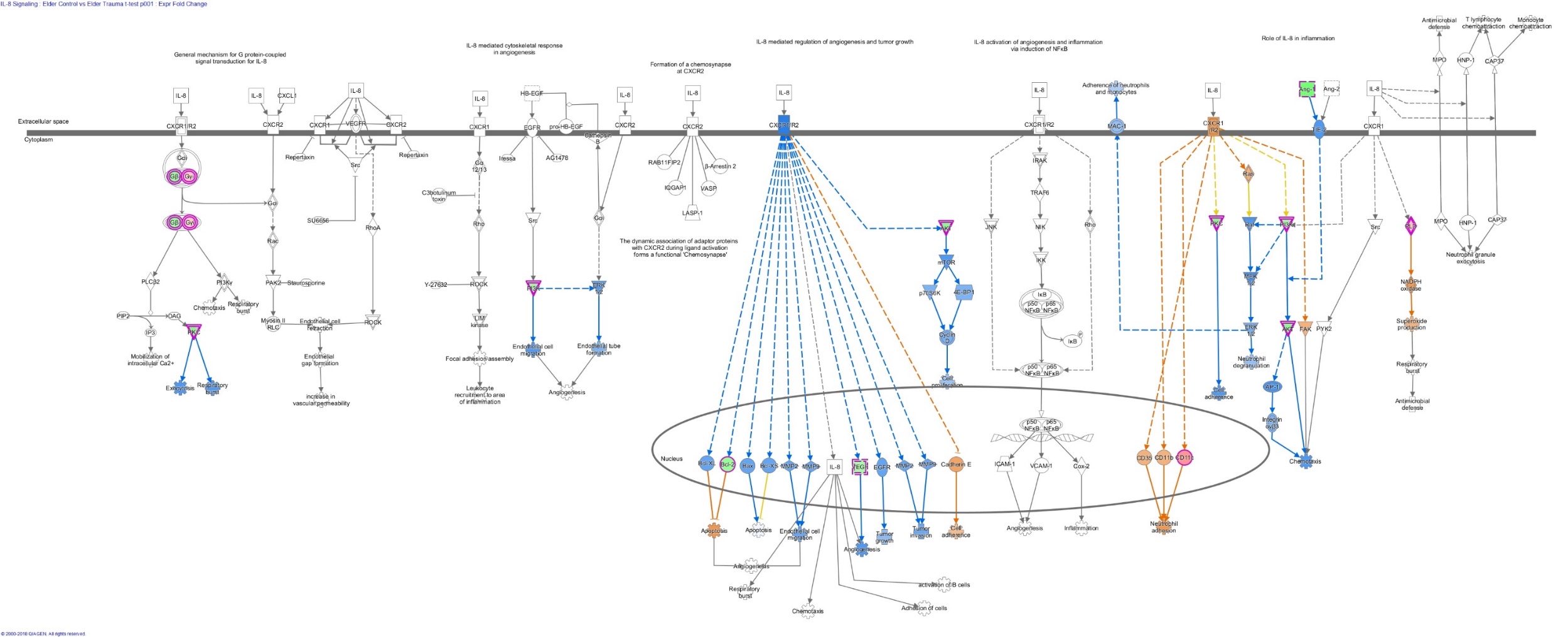 |

**Supplementary Figure 2.** IPA canonical pathway analysis illustration showing differences in the regulation of many differentially expressed genes involved in the IL-8 signaling pathway (important in inflammation) between (**A**) young and (**B**) old adult trauma patients. Orange to red = upregulation, green to blue = downregulation.

**A B**

**
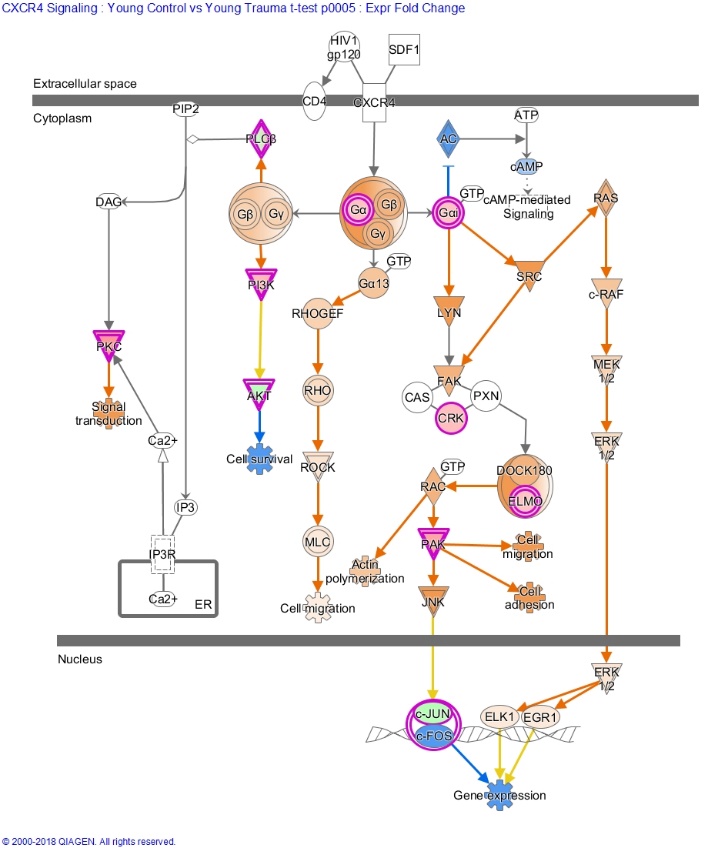

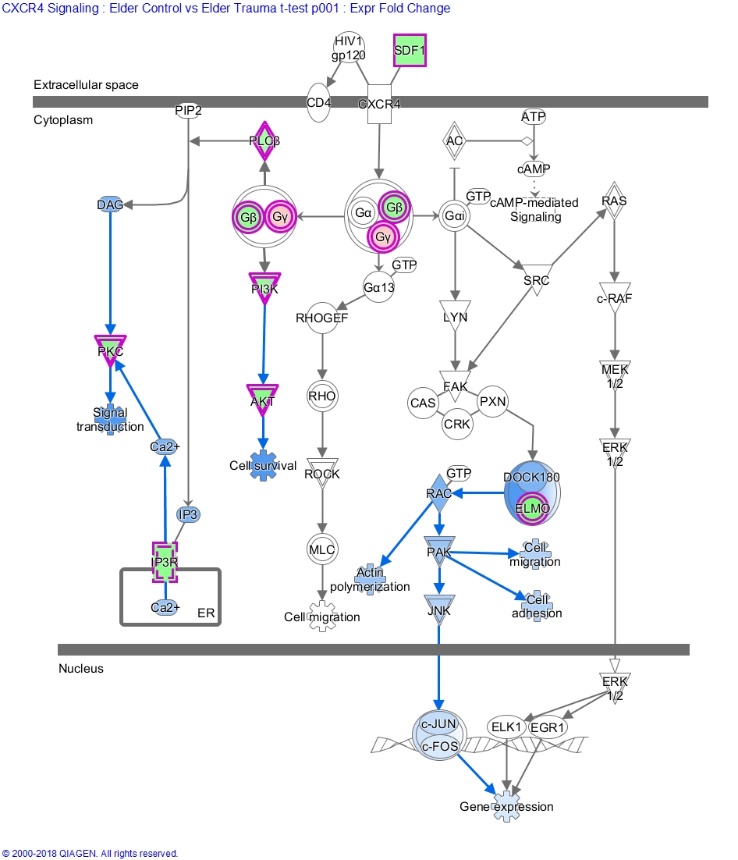
**

**Supplemental Figure 3.** IPA canonical pathway illustration showing differences in the regulation of many differentially genes involved in the CXCR4 signaling pathway between (**A**) young and (**B**) old adult trauma patients. Orange to red = upregulation, green to blue = downregulation.
